# Application of a Novel One-Side Cell Quartz Crystal Microbalance Immunosensor in the Determination of Alpha-Fetoprotein from Human Serum

**DOI:** 10.3390/diagnostics13091630

**Published:** 2023-05-05

**Authors:** Yan Chen, Huashan Shi, Bo Mu

**Affiliations:** 1 School of Information Engineering, Southwest University of Science and Technology, Mianyang 621010, China; 2 Department of Biological Therapy, West China Hospital, Sichuan University, Chengdu 610047, China; shihuashan@scu.edu.cn; 3 School of Basic Medical Sciences, North Sichuan Medical College, Nanchong 637100, China; ppnu2013@163.com

**Keywords:** alpha-fetoprotein (AFP), quartz crystal microbalance (QCM), immunosensor, human serum

## Abstract

The rapid and accurate detection of alpha-fetoprotein (AFP) levels is of great significance for the diagnosis and later treatment evaluation of liver cancer. In this study, a novel integrated quartz crystal microbalance (QCM) immunosensor based on the design to contact liquids on one side only was developed for the label-free detection of AFP. Anti-AFP mouse monoclonal antibodies were immobilized onto the upper electrode surface of the pre-treatment crystals using the staphylococcus aureus protein A. The AFP antigens in human serum were captured by specific surface-coated antibodies, and testing was carried out by monitoring the corresponding changes in frequency. The concentration range of the antigen detected was 13–760 ng/mL. The frequency characteristics of the process of antibody immobilization were investigated in detail, and high reproducibility of AFP antibody immobilization was achieved (standard deviation (SD) = 2.2 Hz). The developed QCM measurement system demonstrated a short test time (only 30 min), good reproducibility (the biological activity can still maintain more than 90% of the first test till it is reused five times), and accuracy as good as the one achieved with the radioimmunoassay (the maximum relative deviation = 4.8%). The designed QCM test system can easily and quickly detect AFP concentrations up to 760 ng/mL, indicating that the developed QCM assay is likely to lead to an alternative approach in large-scale screening for liver cancer in the near future.

## 1. Introduction

Liver cancer is one of the most prevalent cancers in the world [1]. Its mortality rate makes it the second-most deadly cancer in the world, following lung cancer. The core reason for this is the low rate of early diagnosis; nearly 80% of the patients have entered the middle and late stages once found, losing the chance of radical surgical resection. The detection of tumor markers in serum is one of the methods for early diagnosis [2]. Alpha-fetoprotein (AFP), the most specific marker for liver cancer, is traditionally detected by many analytical techniques, including radioimmunoassay (RIA), chemiluminescence immunoassay (CLIA), enzyme-linked immunosorbent assay (ELISA), time-resolved fluorescence immunoassay (TRFIA), etc. [3,4,5,6,7]. Nevertheless, many disadvantages are involved in these techniques, such as the need for a label, radioactive contamination, their time-consuming nature, severe temperature interferences, and the need for expensive instrumentation. Therefore, developing a label-free, safe, rapid, steady, and economical approach to detecting AFP concentrations in serum for large-scale screening of liver cancer is of great importance.

The quartz crystal microbalance (QCM) is a label-free, acoustic bio-sensing system that has become a powerful bioanalytical tool in recent years. To date, QCMs have been widely used in biological and biomedical research [8,9,10,11,12,13,14]. As a shear-mode device, QCM consists of a thin AT-cut quartz disk sandwiched between two gold electrodes. In QCM immunosensors, the biologically active component, such as an antibody, is immobilized onto the surface of the electrode. When the antigen is recognized by the immobilized antibody, through the mass-loading effect of QCM, the specific recognition is transformed into a frequency change that can be measured by a universal frequency counter. Finally, an obvious dose–response correlation can be established between the variations of the analyte concentration and frequency shifts. In the 1950s, Gunter Sauerbrey pointed out the linear relationship between the change in mass and the corresponding resonance frequency shift of the QCM as follows [15]:

(1) $$\Delta f=-\frac{2nf_{0}{}^{2}}{A{\left({\bar{C}}_{66}\rho_{q}\right)}^{\frac{1}{2}}}\Delta m$$

where Δm is the change in the mass deposited on the surface of QCM (in g), Δf is the monitored frequency change, which is caused by the changed mass on the electrode surface (in Hz), $f_{0}$ is the resonant frequency of the first mode (in Hz), *n* is the overtone mode, A is the active vibrating area (in cm^2^), and ${\bar{C}}_{66}=2.947\times 10^{11}\;g\cdot {\mathrm{cm}}^{-1}\cdot {\;s}^{-2}$ and $\rho_{q}=2.648\;g\cdot {\mathrm{cm}}^{-3}$ are the shear modulus and density of quartz, respectively. The negative sign shown in the equation reflects that the frequency of QCM decreases as the deposited mass on the electrode surface increases.

**Figure 1:**
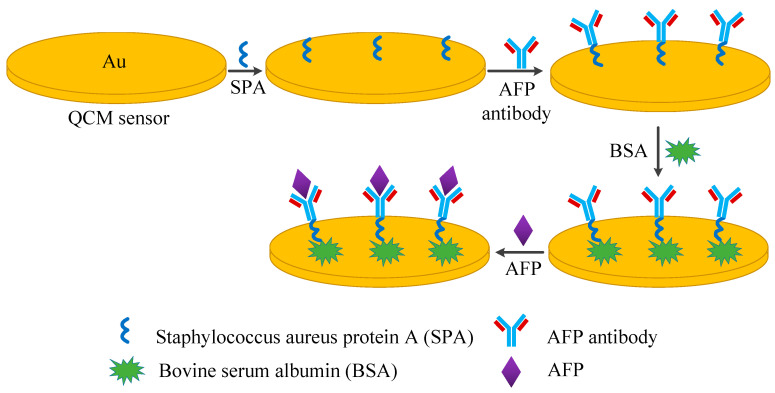
Principle of QCM sensor for the detection of alpha-fetoprotein.

In this work, the QCM immunosensor’s experimental techniques for AFP detection from serum were investigated. We developed a novel QCM measurement system, which consisted of a design to contact serum on one side of the crystal, a homemade Pierce–Gate crystal oscillator with a harmonic suppression circuit (inductance and capacitance combination) to drive the QCM at its third harmonic frequency, and a universal frequency counter for frequency data monitoring. As schematically illustrated in Figure 1, the QCM’s crystal surface was coated with an anti-AFP mouse monoclonal antibody. To expand the potential sites for binding to AFP antigens, AFP antibodies were immobilized using the protein A immobilization technique, and protein A was bound to the Fc segment of the antibody. When the coated anti-AFP bound with AFP in human serum, the changed frequency induced by the change in mass of the anti-AFP/AFP immunocomplex was monitoring within a certain antigen concentration range. Particular attention was given to the detection time and frequency characteristics for the process of antibody immobilization. More details of the analytical performances of the developed QCM sensor were investigated, including the selectivity, linear range, reproducibility, and reusability.

## 2. Materials and Methods

### 2.1. Reagents

Recombinant staphylococcus aureus protein A (SPA, ab52953) was obtained from Abcam (Cambridge, UK). Anti-AFP mouse monoclonal antibody was supplied by Shanghai Tongwei Industrial Co., Ltd. (Shanghai, China). Human AFP antigen was purchased from Cal Bioreagents (Foster City, CA, USA). Serums of cancer patients were provided by West China Hospital of Sichuan University (Sichuan, China). The blocking reagent employed in this study was bovine serum albumin (BSA) from Sigma Co. (Livonia, MI, USA). Phosphate-buffered saline (PBS, pH 7.4) was prepared using 137 mM NaCL, 2.7 mM KCL, 10 mM Na_2_HPO_4_, and 2 mM KH_2_PO_4_. All other reagents used were of the superior grade (over 99.8%, *w*/*w*) to guarantee the trace analysis.

### 2.2. Equipment and Apparatus

In QCM immunosensors, high mass sensitivity is desirable. If the resonator works in its fundamental frequency, it is necessary to increase the frequency of the resonator and thus reduce the thickness of the crystal to obtain high-quality sensitivity. However, the mechanical strength deteriorates as the thickness decreases, resulting in a crystal easily ceasing its oscillation [16,17]. Since overtone resonators have better frequency stability than fundamental resonators at the same resonant frequency, all quartz crystals (10 MHz, optically polished surface, 8 mm in crystal diameter, and 5 mm in gold electrodes diameter) employed in this study were third overtone AT-cut with $35{}^{\circ}16^{\prime }$ angle in order to guarantee high mass sensitivity and good frequency stability. These crystals were obtained from Wintron Inc. (Zhengzhou, China) and were driven by a homemade oscillator circuit.

**Figure 2:**
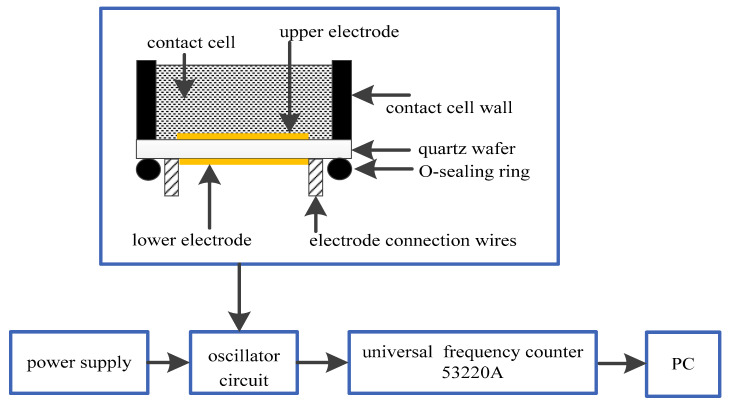
QCM sensor measurement system for detection of the AFP.

**Figure 3:**
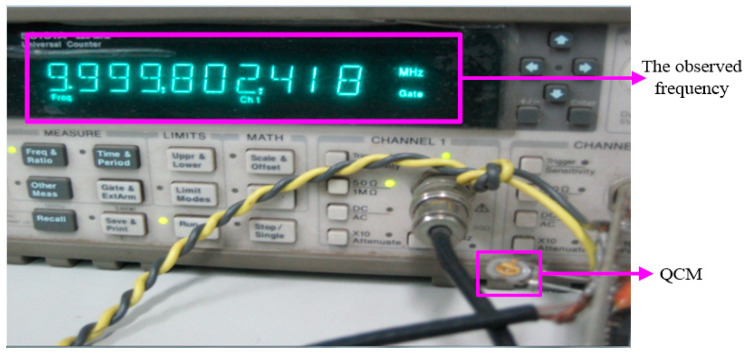
The snapshot of QCM system.

The quartz crystal, the heart of the QCM, was designed to make contact with serum at one side only. This design intended to eliminate the influences of liquid damping and non-uniform coating to allow the crystal reach a stable oscillation condition and ensure the repeatability of detection. Agilent universal frequency counter 53220A, with a resolution of 12 s/bit was used to measure the resonant frequency. The measurement system was shown in Figure 2. Figure 3 shows the snapshot of QCM system.

### 2.3. Antibody Immobilization Procedure

#### 2.3.1. Quartz Crystal Preparation and Pre-Treatment

Two piezoelectric quartz crystals were required in each assay: one was detection crystal and was used to detect AFP; the other was reference crystal and was used to eliminate the non-mass effect and any other system error. In order to prevent the gold electrode from being corroded by chemical solution, the electrical connection of gold electrodes must be firstly sealed with silastic film. After the silicone membrane was completely dry, the gold surfaces of quartz crystals were cleaned by dipping into piranha solution (30% H_2_O_2_:H_2_SO_4_ = 1:3, by volume) for 1 min, and then washed with distilled water, and dried with the steam of nitrogen gas. This cleaning process was repeated three times, ensuring that any possible contaminants were removed before the crystal was loaded while obtaining a hydrophilic gold surface on the crystal surface. Then, the resonance frequency of unloaded and pre-treated QCM was recorded as f_1_. After these preparations, the crystals were used immediately for the immobilization of the antibody. 

#### 2.3.2. Antibody Immobilization

One complete antibody immobilization cycle included the following steps: Firstly, 5 µL of SPA solution (10 mg/mL) in PBS (pH 7.4) was spread on the upper electrode side of the pre-treatment crystals for 30 min. The loaded crystals were thoroughly dried at 25 °C and then the resonance frequency of SPA film-loading QCM was recorded as f_2_. Secondly, 5 µL of anti-AFP monoclonal antibody solution (6 mg/mL) was injected onto electrode surface of the detection, incubated at 22 °C for 2 h, and then followed by PBS and distilled water washing. The resonant frequency f_3_ was recorded after the loaded crystals were thoroughly dried. Finally, the electrode surface was blocked with 50 µL of BSA (20 mg/mL) for 1 h at 4 °C, and washed with PBS. The reference crystal was modified to contain SPA and BSA, but no anti-AFP monoclonal antibody. 

### 2.4. Detection Procedure

To detect AFP, the modified quartz crystals were removed from the 4 °C environment into two different reaction cells, and the operating temperature of the measurement was 25 °C. Then, 100 µL of PBS (pH 7.4) was filled in two reaction cells as the detection buffer solution. After the signal reached steady state, the resonance frequencies of the detection crystal (marked with $F_{0d}$) and reference crystal (marked with $F_{0r}$) were monitored in real time. Finally, 1 mL of AFP standard substance solution containing different concentrations was slowly pumped into the contact wells using a syringe pump. When the immunoreaction between the coated antibody and its homologous antigen was over, the QCM frequency stabilized, and then the steady-state resonance frequencies were real-time online recorded as $F_{1d}$ and $F_{1r}$, respectively. The same test process was applied in the AFP concentration detection of the serum samples from cancer patients.

## 3. Results and Discussion

### 3.1. Concentration Selection for AFP Antibody 

**Figure 4:**
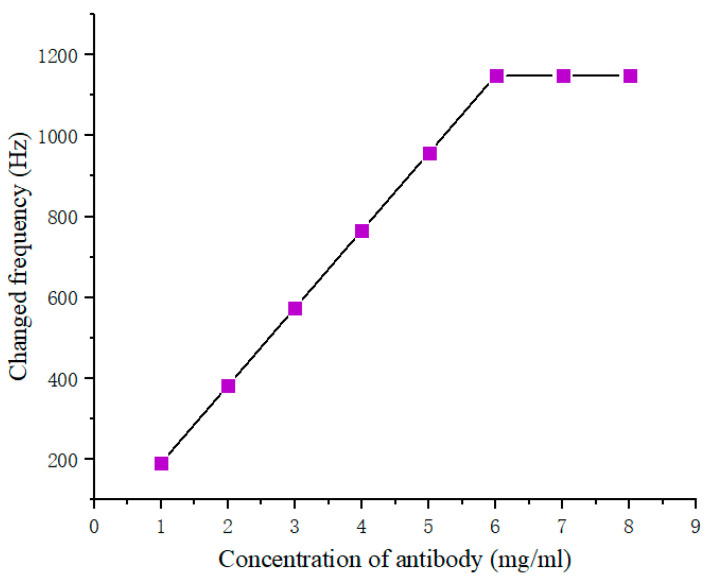
QCM immunosensor response for different concentration of anti-AFP antibody.

The amount of the antibody immobilized on the electrode surface is critical for the QCM assay because has a large influence on the accuracy of the QCM test. In order to provide good performance for the sensor, the anti-AFP antibody pre-titration was performed first. Antibody solutions of 5 µL with different concentrations were deposited on an SPA film layer on each detection crystal. The frequency change upon different antibody concentrations are shown in Figure 4. Within 6 mg/mL, the changed frequency of the immunosensor increased as the deposited antibody concentration increased. Above 6 mg/mL, the frequency did not change, despite the fact that the antibody concentration increased. Therefore, a 6 mg/mL antibody concentration was considered the optimal concentration to be immobilized on the electrode surface in this work, and was thus used for subsequent studies.

### 3.2. Reproducibility of Antibody Immobilization

The reproducibility of antibody immobilization is directly related to the reproducibility of the QCM assay results, thus determining the utility of the QCM immunosensor in clinical laboratories. Since the target antibody to be immobilized is a protein, in this study, protein A was used to immobilize the antibody using the similarity principle. The same AFP standard substance solution was determined by ten different immunosensors repeatedly under optimal conditions. The frequency responses upon antibody immobilization and antigen recognition are both shown in Table 1. These results indicate that good reproducibility of AFP antibody immobilization can be achieved by protein A coating with the standard deviation (SD) within 3 Hz, and the immunosensor has a higher recognition activity when the coefficient of variance is less than 2%. The results demonstrate that the one-side cell QCM method is reliable in AFP determination in human serum. 

### 3.3. Optimal Detection Time of QCM Immunosensor Device

To shorten the total detection time of the assays, the antigen–antibody binding time, which is temperature-dependent, must be investigated. A temperature between 15 and 40 °C is appropriate; within this range, the higher the temperature, the faster the movement of the antigen–antibody molecules; thus, the more collision opportunities, and the faster the binding reaction between the antigen and the antibody. Generally, they bind best at 37 °C [18]. Nevertheless, the operating temperature of the assays cannot be set at 37 °C. Since the environmental temperature has a great influence on the resonant frequency of the quartz crystal, the frequency–temperature characteristics of quartz resonators must be considered in the process of antigen–antibody binding. At 37 °C, the AT-cut quartz resonator used in this study will experience a frequency offset of about 250 Hz [19], which can introduce errors into the test. Fortunately, the temperature dependence of the resonant frequency of the AT-cut is zero at 25 °C [20]; therefore, the detection was carried out in the environment of 25 °C. 

**Figure 5:**
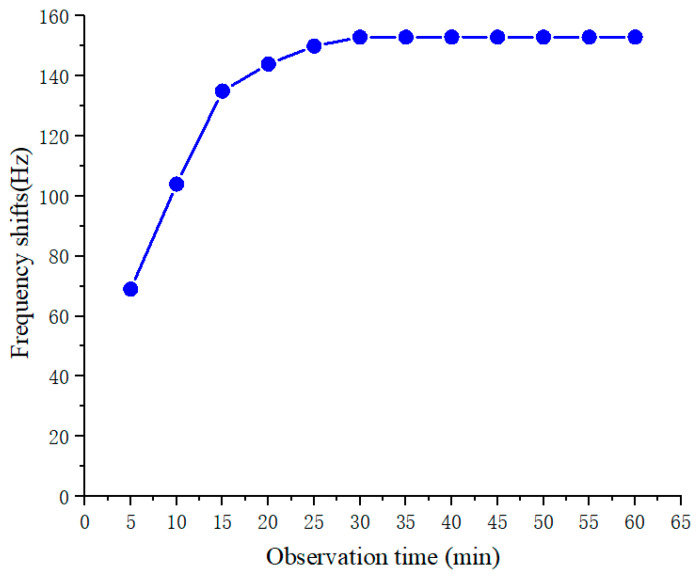
Selection of optimal detection time.

To investigate the optimal time of the antigen–antibody reaction, 400 ng/mL of the AFP standard substance solution was added to the surface of immunosensor, the total observation time was set to 60 min, and the frequency shifts induced by specific adsorption were recorded every 5 min. Within 30 min, with the increase in the immune reaction time, the amount of antigen and antibody binding on the surface of the quartz crystal increased, inducing the frequency shifts of the quartz crystal to increase slowly. When the immune reaction time extended beyond 30 min, the frequency shifts induced by the binding reaction of the antigen and antibody reached an equilibrium, indicating that the immunoreaction on the crystal surface was already over (shown in Figure 5). Therefore, the detection time of 30 min is sufficient to ensure a complete immune reaction between the AFP antibody and its homologous antigen. Thus, the optimal detection time for the immunosensor in this study was 30 min.

### 3.4. Standard Curve for Determination of AFP

**Figure 6:**
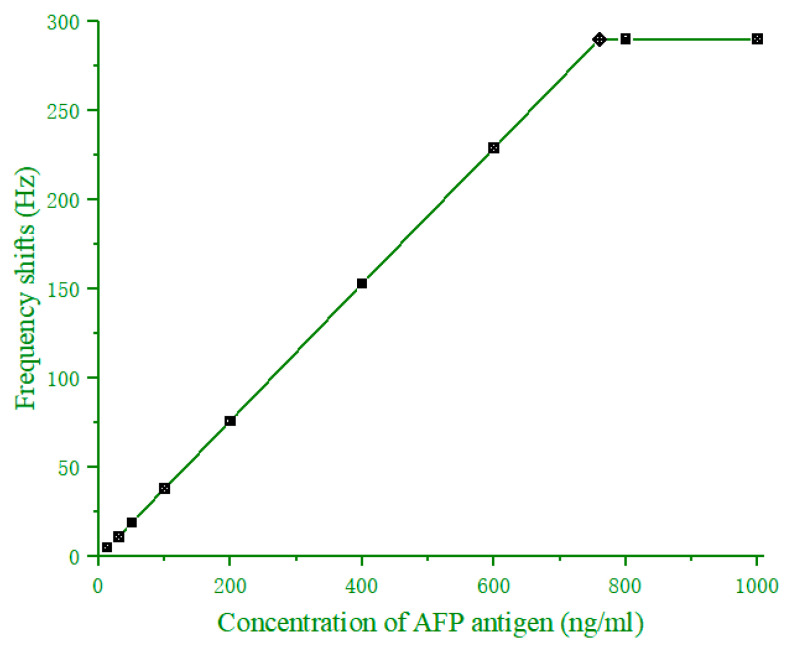
QCM immunosensor response for different concentrations of anti-AFP antibody.

The standard curve method was used for a quantitative analysis of AFP in human serum. At first, incubated various concentrations (0–1000 ng/mL) of the AFP antigen with 6 mg/mL of AFP antibody were coated on the quartz crystal for 30 min. Then, real-time online monitoring of QCM responses was conducted to determine the frequency variation caused by the immune reaction between the AFP antigen and antibody at different concentrations. The reference crystal was employed to eliminate the non-mass effect of immunosensor, which was induced by damping loading of the antigen solution. The frequency shift (ΔF) of the immunosensor was as follows:

(2) $$\Delta F=(F_{1d}-F_{0d})-(F_{1r}-F_{0r})$$

where $\left(F_{1d}-F_{0d}\right)$ and $\left(F_{1r}-F_{0r}\right)$ were the frequency shifts of the detection crystal and reference crystal, respectively. The standard curve is illustrated in Figure 6. The changed frequency of the sensor increased as the AFP antigen concentration increased, and a wide linearity range from 13 to 760 ng/mL was determined. However, beyond 760 ng/mL, saturation was encountered because all the antigen-binding sites in the coated antibodies were exhausted. The appeared antigen excess makes it impossible to accurately detect the AFP level in a serum sample. Thus, when the AFP level in cancer patients’ serum was higher than 760 ng/mL, the serum sample had to be diluted first before another detection was repeated. Moreover, the limit of blank (LOB), the limit of detection (LOD), and the limit of quantitation (LOQ) of the immunosensor were 8.7 ng/mL, 9.6 ng/mL, and 29 ng/mL, respectively.

### 3.5. Selectivity of QCM Sensor 

**Figure 7:**
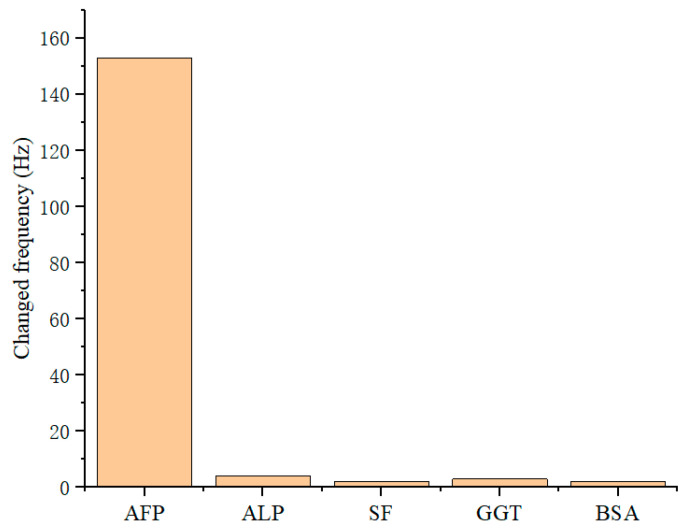
The frequency changes of QCM sensor induced by AFP and different protein solution.

In addition to AFP, the serum samples that were tested often contained a variety of proteins during preparation, such as alkaline phosphatase (ALP), serum ferritin (SF), and gamma-glutamyl transpeptidase (GGT). In addition, a standard bovine serum protein (BSA) was used for site blocking of the sensor. Therefore, to investigate the selectivity of the QCM immunosensor to AFP, a 1 mL PBS solution containing different proteins (AFP, ALP, SF, GGT, and BSA) in a concentration of 400 ng/mL was slowly loaded onto the surface of sensor. The result shows that the frequency change due to the interfering substances produced almost negligible response, indicating that the selectivity of the QCM sensor was acceptable (Figure 7).

### 3.6. Detection of AFP from Real Serum Samples 

A serum sample analysis was carried out in the liquid phase to avoid a tedious dipping procedure. In solution phase sensing, the change in frequency of the QCM immunosensor was governed not only by the viscosity and density of the serum sample, but also by other non-mass serum parameters, such as conductivity, solution polarity, temperature, and the thickness of the serum solution film upon the crystal [15,21,22,23,24]. To eliminate the non-mass loading effect and any errors caused by other non-loading effects, the frequency response be obtained from reference crystal must be removed from the total frequency response from the detection crystal. To investigate the feasibility of the QCM immunosensor for possible applications in the early detection of liver cancer, the AFP level was detected in real serum samples obtained from eight cancer patients (one lung cancer patient, one ovarian cancer patient, and six liver cancer patients), and the results are shown in Table 2. In addition, the serum samples were also analyzed by radioimmunoassay (RIA), which is a practical technique for serum analysis used across the world. Table 2 demonstrates good agreement between the two methods, with relative deviations below 5%. 

Other comparisons of major characteristics of the QCM and the RIA were conducted, as shown in Table 3, indicating that the QCM is superior to RIA in terms of the detection time, safety, cost, and convenience. Therefore, the designed QCM immunoassay has a broad application prospect in the clinical laboratory diagnosis and analysis of AFP.

### 3.7. Reproducibility of QCM Immunosensors 

**Figure 8:**
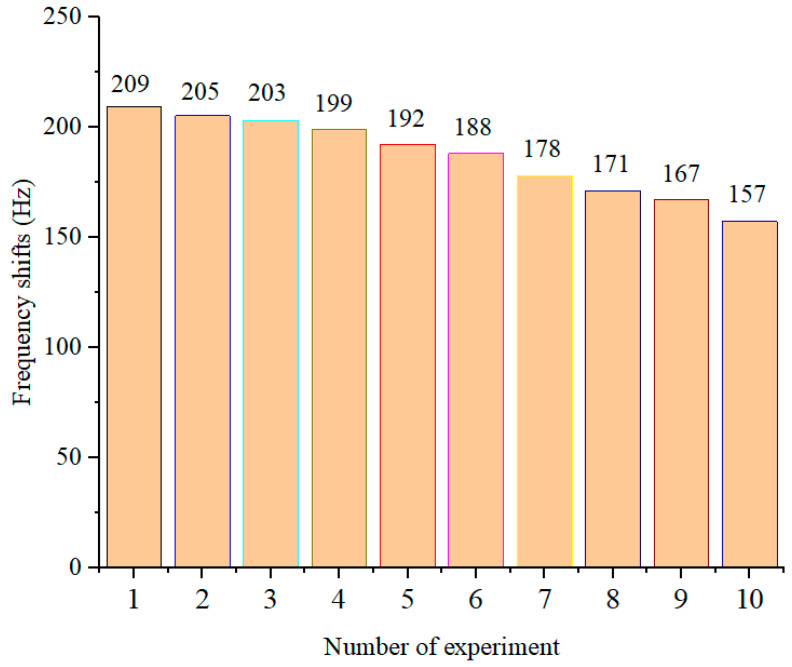
QCM immunosensor frequency response for the reused times.

Lastly, the reproducibility of the QCM immunosensor was investigated. Usually, reproducibility is the most basic parameter used to evaluate the performance of the sensor and also one of the key factors used to determine whether the sensor can be used in clinical diagnosis. Two aspects of reproducibility were covered. One of these aspects is the repeatability of multiple detections of the same sensor on the same serum sample, which is also known as the reusability of the sensor. The other aspect is the repeatability of the detection results of different sensors on the same serum sample. In this study, the 8# sensor and 8# serum sample in Section 3.5 were employed for the investigation into reusability. After the first test, the 8# sensor was immersed in a solution containing 1.2 M sodium hydroxide and 7 M urea for 10 min to denature the anti-AFP/AFP immunocomplex, followed by rinsing with double-distilled water and a PBS buffer solution. After cleaning with ultrasonic in deionized water, the regenerated QCM sensor could be reused to conduct a new experimental analysis, and the effect of reusability is shown in Figure 8. With the increasing reuse times, the change in frequency due to the same sample decreased slowly, and the frequency change upon the sixth test was down to 90% of the first measurement. Therefore, a QCM immunosensor could be reused six times without bringing large errors into the experiments. Meanwhile, the 8# serum sample was investigated by ten different QCM immunosensors and the results are shown in Table 4, indicating that different QCM immunosensors with the same preparation method and the same detection procedure can offer reliable results for the test of AFP levels in human serum.

## 4. Conclusions

The rapid and convenient determination of AFP levels is essential for early liver cancer diagnoses. It gives the patients the chance to have a radical surgical resection and dramatically improve the survival rate of patients. In this study, a QCM immunosensor based on the design to contact serum on one side only was successfully developed for the determination of AFP from serum samples that were obtained from cancer patients. The developed QCM measurement system offers good performance in terms of simplicity, analysis time, specificity, accuracy, reproducibility, and security, and it achieved the detection of AFP at concentrations of 13–760 ng.mL^−1^. It is necessary to further improve the measurement system to expand the detection concentration range of AFP, and the analytical systems need to be optimized to reach clinical application. As an effective diagnostic method for the detection of serum AFP in a clinical laboratory in the near future, the QCM-based analytical method is undoubtedly promising.

## Figures and Tables

**Table 1:** Reproducibility of AFP antibody immobilization and the corresponding specific recognition activity of QCM immunosensor.

Crystal Number	AFP Antibody Immobilization Δf (Hz)	^a^ Antigen Recognition Δf (Hz)
1	1147	150
2	1148	156
3	1142	151
4	1146	152
5	1149	155
6	1144	158
7	1147	153
8	1143	154
9	1146	152
10	1148	159
Average	1146	154
SD	2.2	2.82
CV(%)	0.19	1.8

^a^ The concentration of antigen is 400 ng/mL.

**Table 2:** Comparison between QCM and RIA method for diagnosis AFP levels.

Serum Samples	1 ^a^	2 ^b^	3 ^c^	4 ^c^	5 ^c^	6 ^c^	7 ^c^	8 ^c^
QCM number	**1**	**2**	**3**	**4**	**5**	**6**	**7**	**8**
QCM (ng.mL^−1^)	15.6	18.2	423.4	289.5	355.8	199.3	501.1	545.3
RIA (ng.mL^−1^)	15.2	17.7	436.1	280.3	342.4	190.9	517.1	520.5
Relative deviation (%)	2.6	2.8	−2.9	3.3	3.9	4.4	−3.1	4.8
correlation coefficient (r)	0.9978							

^a^ Serum sample from lung cancer patient. ^b^ Serum sample from ovarian cancer patient. ^c^ Serum samples from liver cancer patients.

**Table 3:** Main characteristics of the two methods in the determination of AFP.

Method	Analytic Time	Radio Contamination	Labeled Antibody	Cost	Analysis Process	References
QCM	Half hour	No	No	Low	Simple	This study
RIA	Hours	Yes	Yes	High	Cumbersome	[4]

**Table 4:** Frequency response for the same serum sample with different QCM immunosensor under the same condition.

QCM Numbers	1	2	3	4	5	6	7	8	9	10
Frequency changes (Hz)	209	207	203	211	205	206	210	208	204	209
Average (Hz)	207									
SD (Hz)	2.5									

## Data Availability

The datasets generated for this study are available on request to the corresponding author.
